# Lung cancer incidence among world trade center rescue and recovery workers

**DOI:** 10.1002/cam4.4672

**Published:** 2022-03-28

**Authors:** Keith Sigel, Rafael E. de la Hoz, Steven B. Markowitz, Chung Yin Kong, Kimberly Stone, Andrew C. Todd, Juan P. Wisnivesky

**Affiliations:** ^1^ Division of General Internal Medicine Icahn School of Medicine at Mount Sinai New York New York USA; ^2^ Department of Environmental Medicine and Public Health Icahn School of Medicine at Mount Sinai New York New York USA; ^3^ Earth and Environmental Sciences Queens College, City University of New York Queens New York USA

**Keywords:** lung cancer, lung diseases, occupational diseases, occupational lung disease, respiratory diseases

## Abstract

**Background:**

Many World Trade Center disaster (WTC) rescue and recovery workers (WTC RRWV) were exposed to toxic inhalable particles. The impact of WTC exposures on lung cancer risk is unclear.

**Methods:**

Data from the WTC Health Program General Responders Cohort (WTCGRC) were linked to health information from a large New York City health system to identify incident lung cancer cases. Incidence rates for lung cancer were then calculated. As a comparison group, we created a microsimulation model that generated expected lung cancer incidence rates for a WTC‐ and occupationally‐unexposed cohort with similar characteristics. We also fitted a Poisson regression model to determine specific lung cancer risk factors for WTC RRWV.

**Results:**

The incidence of lung cancer for WTC RRWV was 39.5 (95% confidence interval [CI]: 30.7–49.9) per 100,000 person‐years. When compared to the simulated unexposed cohort, no significant elevation in incidence was found among WTC RRWV (incidence rate ratio [IRR] 1.34; 95% CI: 0.92–1.96). Predictors of lung cancer incidence included age, smoking intensity, and years since quitting for former smokers. In adjusted models evaluating airway obstruction and individual pre‐WTC occupational exposures, only mineral dust work was associated with lung cancer risk (IRR: 2.03; 95% CI: 1.07–3.86).

**Discussion:**

In a sample from a large, prospective cohort of WTC RRWV we found a lung cancer incidence rate that was similar to that expected of a WTC‐ and occupationally‐unexposed cohort with similar individual risk profiles. Guideline‐concordant lung cancer surveillance and periodic evaluations of population‐level lung cancer risk should continue in this group.

## Graphical abstract

In this cohort study of 17,668 World Trade Center rescue and recovery workers the risk of lung cancer was not increased compared to expected rates estimated by a detailed microsimulation model. The World Trade Center disaster has not yet had a major impact on lung cancer risk for exposed persons.

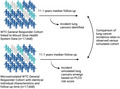

## 1 INTRODUCTION

The September 11, 2001, terrorist attacks and subsequent World Trade Center (WTC) collapse exposed thousands of persons to a poorly characterized toxicant mix, possibly including carcinogens. During a 9‐month rescue and recovery effort, many workers and volunteers (hereafter WTC RRWV) were exposed to substantial doses of these toxicants.^1^ As a result, acute and chronic respiratory symptoms have been well described in this cohort, as have abnormalities found on chest imaging.^2^, ^3^, ^4^, ^5^ The impact of exposure to these substances on lung cancer risk, however, has been insufficiently studied.

The potential impact of carcinogenic environmental exposures during the disaster and its aftermath have received significant attention, and cancer risk has been a key concern in the WTC responder cohort.^6^, ^7^ The evaluation of lung cancer incidence in WTC responders is complex, however, as occupational exposures were not adequately measured at the disaster site, and this heterogeneous population had varied cigarette smoking exposure as well as a wide range of pre‐WTC occupational exposures that could also have strongly influenced their cancer risk.

Earlier analyses using the World Trade Center Health Registry's linkage to New York State cancer registry data (through 2008) did not find an excess of lung cancers among WTC‐exposed persons.^8^ Similar contemporaneous studies of New York City firefighters^9^ and of general responders^6^ reported a lower risk of lung cancer than expected based on population rates. However, these studies did not consider individual‐level lung cancer risk factors in their analyses, and all of them took place a relatively short time after the exposure of interest, compared to the expected long latency of lung cancer. Moreover, these studies compared lung cancer rates among WTC RRWV to general population controls, which are a less than optimal comparator group due to the unique demographic and behavioral characteristics of these cohorts, and the well‐known “healthy worker bias” in occupational cohorts.^10^

To clarify the risk of lung cancer in this WTC general responder cohort, we used data from the WTC Health Program (WTCHP) General Responder Cohort (WTCGRC) linked to information from the electronic health records of our health care system to estimate lung cancer incidence and risk factors among WTC general responders.

## 2 METHODS

We used data from the WTCGRC linked to electronic medical records from the Mount Sinai Health System (MSHS) during 2002–2018. This study was approved by the Mount Sinai Program for the Protection of Human Subjects (HS18‐00583). All subjects were consented participants in the WTCGRC, a prospective screening, surveillance, and treatment (for conditions certified as WTC‐related) program for workers of all occupations (except firefighters, who have their own branch of the WTCHP) who participated in the rescue, cleanup and/or service restoration of the WTC disaster sites. The make‐up and enrollment of this cohort have been previously reported.^11^ Starting in 2002, WTCGRC participants underwent standardized baseline examinations that included assessment of WTC exposure, pre‐WTC occupational exposures, and smoking history, as well as spirometry and chest radiography. Surveillance included periodic follow‐up visits. In this study, we included only persons with linked electronic data and follow‐up time within the MSHS (*n* = 17,668). As lung cancer or related symptoms may have led persons to seek care in the WTCHP, we excluded people with lung cancers diagnosed within the first 3 months of joining the WTCHP or with the report of prior lung cancer (*n* = 19).

We collected demographic information (age, sex, race, and ethnicity) and self‐reported educational attainment from the WTCHP database. We used questionnaire information to determine smoking status, as well as pack‐year exposure (using average cigarettes per day and smoking duration in years), and for former smokers, we calculated the number of years since quitting smoking. Participants were classified as non‐smokers if they had smoked less than 20 packs of cigarettes or less than one cigarette per day for up to 1 year. We also ascertained baseline body mass index (BMI) and evidence of chronic obstructive pulmonary disease (COPD) diagnosis (from linked diagnostic data and/or self‐report). For participants with available spirometry data, we calculated forced expiratory volume in 1 s (FEV_1_) to forced vital capacity (FVC) ratio values, categorizing ratios of 0.70 or less as indicative of airway obstruction.

We included a previously published, composite WTC exposure measure based on work duration, time in contact with the debris cloud, and time spent working on the collapsed pile.^3^ This variable was categorized as high (by combining the original very‐high and high categories), intermediate or low. We also included a variable that indicated starting work on the effort within 48 h of the attack. Pre‐WTC occupational exposures were categorized based on self‐report of work‐related contact with vapors, dusts, fumes, and gases from “a few times per week” to “daily.”^4^ These exposures included: asbestos: cadmium; diesel and non‐diesel exhaust; general, mineral, and silica/sand dust; wood dust; fiberglass; industrial cleaning solutions; welding fumes.

Our primary outcome of interest was incident lung cancer. We identified lung cancers via three mechanisms: (1) from WTCGRC data, as a reportable condition; (2) using linked diagnostic code data (ICD9: 162.x; ICD10: C34.x) from MSHS electronic records; (3) using linked cancer registry information from the MSHS cancer registry. We then verified each lung cancer and diagnosis date by manual chart review.

## 3 STATISTICAL ANALYSIS

We first compared baseline characteristics of our sample between those with and without a lung cancer diagnosis, using the Wilcoxon test for continuous and ordinal variables and the chi‐squared test for dichotomous or nominal categorical variables. We then calculated incidence rates for lung cancer, first for the overall sample, then stratified by baseline smoking status. We used Poisson methods to estimate 95% confidence intervals (95% CI) for these rates. To account for our exclusion of prevalent cancer diagnosed in the first 3 months of analytic sample inclusion, we calculated person‐time of follow‐up from 3 months after cohort inclusion date until the last clinical encounter in the MSHS, as there may not have been ascertainment of lung cancer after that date. Using the standard year 2000 United States population (census.gov), we then determined age‐standardized incidence rates overall and for each stratum, with 95% confidence intervals calculated using the gamma distribution.^12^

**FIGURE 1:**
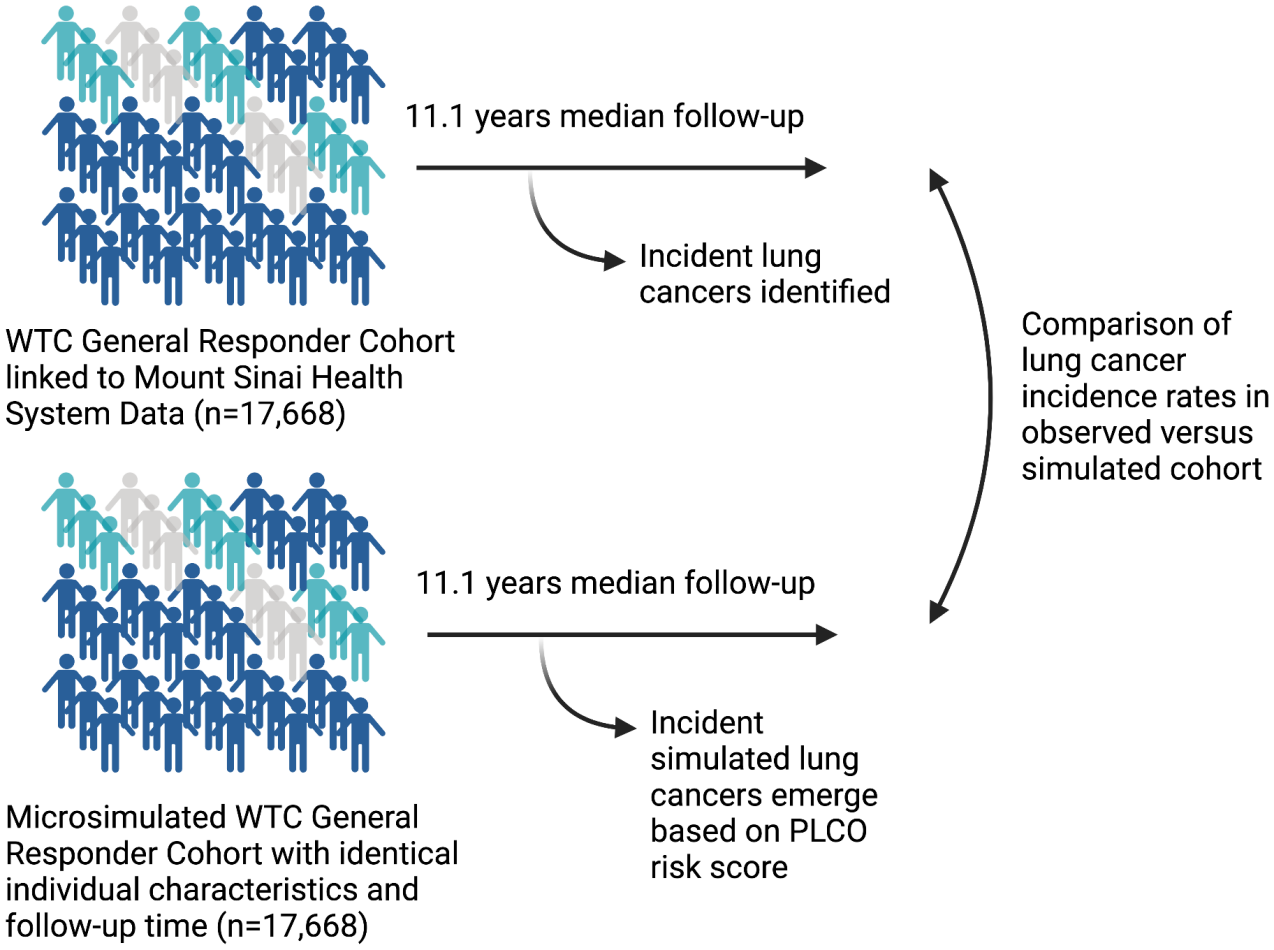
Schematic of study illustrating World Trade Center rescue and responder worker cohort and identical microsimulation comparator cohort

We then employed microsimulation methods to create a comparison group with identical baseline characteristics and lung cancer risk factors as the study sample but with no WTC or other occupational exposures. After assembling the identical cohort, we first calculated 6‐year lung cancer probabilities for each simulated cohort participant, using the PLCO_m2012_ model.^13^ This model, based on data from the large Prostate, Lung, Colorectal, and Ovarian screening randomized control trial, estimates 6‐year lung cancer probability based on age, sex, educational attainment, family history of lung cancer, BMI, smoking status, intensity, and duration, years since quitting smoking, COPD and personal cancer history. WTCGRC data contained all of these variables except the family history of lung cancer, which we randomly imputed using published estimates.^14^ Missing values for BMI for 1.1% of participants were also imputed, using a multivariable linear regression model (see Table S2). Pack‐year smoking was missing for 5% of the study sample; for participants with missing data in the microsimulation, we randomly assigned a pack‐year value from an exponential probability distribution that matched the overall distribution in the WTCHP data for current and former smokers. Six‐year lung cancer probabilities were then calculated for each cohort member, after which all variables were time‐updated to calculate 12‐ and 18‐year probabilities. Current smokers were assumed to continue smoking at the same intensity, and former smokers were assumed to continue their cessation of smoking. Overall probabilities were then converted to monthly probabilities by assuming a constant rate, and microsimulation was performed on each cohort member, using their actual follow‐up time, to create a replica cohort of the observed data, thus estimating an expected number of lung cancer cases (see Figure 1 for schematic). The cohort was simulated 30 times to generate an average number of incident lung cancer cases. We compared the incidence of lung cancer among the WTC sample to the incidence in the simulated cohort by calculating an incident rate ratio for the observed versus simulated groups, estimating 95% CIs using Poisson methods.

Last, we used observed data to fit a Poisson model predicting lung cancer incidence, adjusting for the lung cancer risk factors included in the PLCOm2012 model (except for family history of lung cancer which was unavailable). We evaluated WTC‐ and non‐WTC‐related occupational exposures in a combined model, to determine the relative impact of these factors on lung cancer risk. We also fitted separate, adjusted models, including spirometry data as well as each self‐reported pre‐WTC occupational exposure as an individual exposure. We conducted secondary analyses using multiple imputations to address missing data on education (3%) and pack‐years of smoking (5%). We used multiple imputations with chained equations methods to impute missing values.^15^ The multiple imputation results did not differ substantially from complete case analyses, so we include results from the imputed analyses. Analyses were conducted using SAS 9.3 and STATA 16; the analysis code is available on github (keithsigel‐lab/WTC‐lung‐cancer).

## 4 RESULTS

We identified 70 incident lung cancers cases in the WTCHP‐MSHS‐linked cohort. Lung cancers were more common among older persons (median age at lung cancer diagnosis was 62) and more frequent among persons with less educational attainment, but they did not differ in proportion by sex or race/ethnicity (Table 1). More than 75% of those diagnosed with lung cancer were current or former smokers. Lower BMI was also significantly associated with incident lung cancer (*p* = 0.02) as was increased airway obstruction, measured by the ratio of FEV_1_ to FVC (*p* <0.001). WTC exposure, using a previously published categorical exposure variable, was not significantly associated with lung cancer (*p* = 0.34). Self‐reported pre‐WTC occupational exposures to lung cancer risk factors were frequent (Table 2). Self‐report of previous asbestos, silica, or diesel fumes exposures were not associated with lung cancer, while self‐report of frequent exposure to cadmium and mineral dusts were more common in lung cancer patients than in participants who did not develop lung cancer (both *p* <0.05).

**TABLE 1:** Characteristics for WTC general responders who developed lung cancer and those that did not

Characteristic	No lung cancer	Lung cancer	*p*‐value
Number (%)	17,598 (99.7)	70 (0.4)	
Age at WTCHP enrollment, median (IQR)	44 (38–51)	54 (46–59)	<0.001
Female, *n* (%)	2805 (15.9)	9 (12.9)	0.48
Ethnicity/Race, *n* (%)			0.06
Non‐Latino White	9125 (51.9)	49 (70.0)	
Non‐Latino Black	2072 (11.8)	8 (11.4)	
Latino	2003 (11.4)	4 (5.7)	
Asian	209 (1.2)	0 (0)	
Multiracial	2515 (14.3)	5 (7.1)	
Other	1674 (9.5)	0 (0)	
Educational level, *n* (%)			0.08
<High school	1442 (8.2)	6 (8.6)	
High school graduate	3630 (20.6)	19 (27.1)	
Some college	6741 (38.3)	31 (44.3)	
College graduate	3467 (19.7)	6 (8.6)	
Graduate school	1683 (9.6)	3 (4.3)	
Unknown	635 (3.6)	5 (7.1)	
Smoking status, *n* (%)			<0.001
Current	2420 (13.8)	35 (50.0)	
Former	4300 (24.4)	18 (25.7)	
Never	10,878 (61.8)	17 (23.6)	
Smoking pack‐years for smokers, median (IQR)	7.5 (1.39–18)	27 (0.3–39.9)	<0.001
Body Mass Index, median (IQR)	29.1 (26.4–32.4)	27.6 (25.2–31.3)	0.03
Obstructive lung disease^a^, *n* (%)	1112 (6.7)	15 (24.6)	<0.001
WTC exposure level, *n* (%)			0.24
Low	2469 (14.4)	15 (21.4)	
Intermediate	10,949 (62.2)	40 (57.1)	
High/very high	3737 (21.2)	14 (20.0)	
Missing	443 (2.5)	1 (1.4)	
WTC arrival within 48 h, *n* (%)	11,172 (64.0)	41 (58.6)	0.34
WTC exposure duration, *n* (%)			0.63
≤60 days	5766 (34.4)	26 (37.1)	
>60 days	10,992 (65.6)	44 (62.9)	
Missing			
Follow‐up, years, median (IQR)	11.1 (6.3–14.3)	11.5 (8.4–14.3)	0.13

^a^ defined as FEV1/FVC <0.7; missing spirometry values for 1090 participants.

**TABLE 2:** Self‐reported pre‐WTC occupational exposures by lung cancer status

Characteristic	No lung cancer	Lung cancer	*p*‐value
Number (%)	17,598 (99.7)	70 (0.3)	
Any moderate‐significant occupational exposure, *n* (%)	12,509 (71.1)	48 (68.6)	0.64
Asbestos, *n* (%)	2239 (12.7)	11 (15.7)	0.45
Cadmium, *n* (%)	359 (2.0)	4 (5.7)	0.03
Diesel fumes, *n* (%)	5523 (31.4)	26 (37.1)	0.30
Non‐diesel industrial fumes, *n* (%)	4203 (23.9)	10 (14.3)	0.06
General dust exposure, *n* (%)	10,897 (61.9)	44 (62.9)	0.87
Mineral dust, *n* (%)	1200 (6.8)	12 (17.1)	0.001
Wood dust, *n* (%)	3113 (17.7)	18 (25.7)	0.09
Silica dust, *n* (%)	2730 (15.5)	16 (22.9)	0.09
Fiberglass, *n* (%)	1918 (10.9)	12 (17.1)	0.10
Industrial, *n* (%)	2807 (16.0)	15 (21.4)	0.21
Welding, *n* (%)	1648 (9.4)	11 (15.7)	0.07

The incidence of lung cancer for the study sample was 39.5 (95% confidence interval [CI]: 30.7–49.9) cases per 100,000 person‐years (p‐y) (Table 3) and the age‐standardized rate was 51.4 (95% CI: 40.6*–*112.7) per 100,000 p‐y. Rates for never, former and current smokers were 15.8 (95% CI: 9.2–25.2), 42.1 (95% CI: 25.0–66.5) and 130.2 (95% CI: 90.7–181.1) per 100,000 p‐y, respectively. When compared to the simulated unexposed cohort, there was no significant elevation in the incidence rate among these WTC RRWV (incidence rate ratio [IRR] 1.34; 95% CI: 0.92–1.96). Current smokers did not have significantly higher lung cancer rates, compared to the simulated data while former smokers in the observed cohort had significantly lower lung cancer rates than predicted in the simulation (IRR 0.36; 95% CI: 0.20–0.61).

Significant predictors of lung cancer incidence included age (IRR 1.10; 95% CI: 1.08–1.13), pack‐years of smoking for former and current smokers (IRR 1.03; 95% CI: 1.02–1.04), and years since quitting for former smokers (0–14 years quit, IRR 0.34; 95% CI: 0.15–0.74; 15+ years quit, IRR 0.47; 95% CI: 0.22–0.97) (Table 4). Neither pre‐WTC nor WTC occupational exposure levels were significantly associated with lung cancer risk. In separate adjusted models evaluating airway obstruction and individual pre‐WTC occupational exposures obstructive lung disease from spirometry measurements and self‐reported frequent occupational mineral dust exposure were both independently associated with lung cancer risk (Table S1; obstructive lung disease: IRR 1.97; 95% CI 1.04–3.75, mineral dust exposure: IRR 2.03; 95% CI: 1.07–3.86).

## 5 DISCUSSION

In this sample of WTC general responders, we found that lung cancer incidence was similar to expected rates in a simulation based on a well‐established lung cancer risk model. While most lung cancer risk was associated with established risk factors, such as age and smoking, non‐WTC occupational exposure was also associated with increased risk (Tables 3 and 4).

**TABLE 3:** Lung cancer incidence

Incidence rate	Cases per 100,000 person‐years	95% CI^*^
Whole analytic cohort, observed	39.5	30.7–49.9^**^
Whole analytic cohort, observed, age‐standardized	51.4	40.6–112.7^***^
Whole analytic cohort, simulated	29.4	22.0–38.6^**^
Never smokers, observed	15.8	9.2–25.2^**^
Never smokers, observed, age‐standardized	17.9	7.8–41.0^***^
Never smokers, simulated	6.7	2.9–13.3^**^
Former smokers, observed	42.1	25.0–66.5^**^
Former smokers, observed, age‐standardized	21.7	12.6–103.3^***^
Former smokers, simulated	116.6	90.9–147.3^**^
Current smokers, observed	130.2	90.7–181.1^**^
Current smokers, observed, age‐standardized	403.6	83.8–1010.0^***^
Current smokers, simulated	178.7	138.7–226.5^**^

Incident rate comparisons	Incidence rate ratio	95% CI^*^
Observed cohort versus simulated cohort, all participants	1.34	0.92–1.96^**^
Observed cohort versus simulated cohort, never smokers	2.34	0.96–6.27^**^
Observed cohort versus simulated cohort, former smokers	0.36	0.20–0.61^**^
Observed cohort versus simulated cohort, current smokers	0.73	0.47–1.11^**^

* 95 CI, 95% confidence interval.

** Based on binomial distribution.

*** Based on gamma distribution.

**TABLE 4:** Multivariable model predicting lung cancer incidence

Characteristic	Incidence rate ratio	95% CI
Age	1.10	1.08–1.13
Female sex	1.16	0.55–2.44
Race/ethnicity		
White	Reference	
Black	0.68	0.31–1.46
Latino	0.49	0.17–1.39
Multiracial	0.47	0.18–1.22
Other	0.74	0.26–2.09
Education		
<High school	Reference	
High school graduate	1.21	0.48–3.06
Some college	1.65	0.66–4.12
College graduate	0.66	0.21–2.09
Graduate school	0.36	0.09–1.45
Pack‐years smoking	1.03	1.02–1.04
Former smoker, 0–14 years quit	0.34	0.15–0.74
Former smoker, 15+ years quit	0.47	0.22–0.97
Previous cancer history	0.44	0.16–1.23
Body mass index	0.95	0.90–1.01
High‐risk occupational exposure	1.82	0.97–3.38
WTC exposure level		
Low	Reference	
Intermediate	0.61	0.33–1.11
High/very high	0.62	0.29–1.31

Our study is one of the first to account for the distribution of lung cancer risk factors within a WTC‐exposed cohort to compare lung cancer risk to expected rates. Previous studies have compared observed crude incidence rates to population‐based rates, generally finding similar or lower than expected lung cancer rates among WTC‐exposed workers. Using data from 2005 to 2008, a study of WTC RRWV in the WTC Health Registry reported a lung cancer incidence rate of 31.9 per 100,000 person‐years, non‐statistically significantly lower than the observed rate in the general population of New York state (49.0 per 100,000 p‐y).^8^ A later analysis using WTC Registry data (a cohort of general responders, residents of lower Manhattan, and employees of businesses in the World Trade Center area) found an incidence rate of 35.7 lung cancers per 100,000 person years during the period 2007–2011, significantly lower than population estimates based on Surveillance, Epidemiology and End‐Results registry data (standardized incidence ratio [SIR] 0.69; 95% CI 0.50–0.93).^16^ Smoking prevalence in WTCGRC was lower than the general United States (US) population (14% current smoking for WTCGRC versus 23% for the US in 2002) and these analyses did not control for smoking rates or other important risk factors that might have been substantially different in the WTC cohorts compared to the general population.^17^ In contrast, our study found a similar overall incidence compared to expected rates, after carefully accounting for other, well‐established lung cancer risk factors.

Although incidence rates in our cohort among never smokers were higher (albeit non‐significantly) than predicted by our simulation, they were generally similar to age‐standardized estimates from other large studies.^18^ The PLCO risk score that we used for our comparison group did not consider occupational risk, and a substantial proportion of never smokers in the WTC worker cohort had these exposures. WTC‐related exposures or pre‐WTC occupational exposures may play a role in elevating lung cancer risk that may only be apparent in the never‐smoking sub‐cohort and is obscured among smokers due to a more powerful role of cigarette smoking in determining lung cancer risk. Furthermore, exposure to inhaled carcinogens during the WTC response may have not yet had a substantial impact on lung cancer risk due to latency associated with these types of exposures. It is unlikely that the incidence rates seen in the WTCGRC cohort are impacted by missed cancers as the cohort receives periodic medical surveillance and cancer care coverage. In addition, lung imaging (often using computerized tomography [CT]) has been frequent in this cohort^4^ and, in more recent years, CT‐based lung cancer screening for eligible smokers has been available to cohort members, adding additional levels of early detection and surveillance. It should be noted, however, that the impact of screening on observed lung cancer incidence is likely to be small as this only occurred in the last 2–3 years of the follow‐up period for the current analysis. Nonetheless, as the WTC responder cohort ages, and potential lung carcinogenic exposure latency increases, increasing the incidence of lung cancer is likely.^19^ Accordingly, and as our analyses covered data up to 17 years post‐WTC exposure, we report more incident cases than the previous report from this cohort.^5^ Lastly, epidemiologic evaluations of occupational exposures tend to have modest lung cancer risk increases compared to smoking, supporting a need for continued surveillance and re‐evaluation of risks for WTC RRWV.^20^

Predictors of lung cancer in WTC responders were largely established risk factors; age, smoking intensity, and time since smoking cessation was the most prominent in multivariable analysis. The risk associated with pre‐WTC occupational exposures was limited, although frequent mineral dust exposure was a significant independent risk factor. This occupational category (described as “mineral or mining dusts” to participants in the WTCHP survey) may represent a broad range of exposures in the WTC cohort who consist principally of workers in protective services; construction; buildings and grounds cleaning and maintenance and electrical, telecommunications and other installation and repair groups, possibly including silica.^6^

The overall incidence of lung cancer among WTC responders who were current or former smokers was not less than predicted. Furthermore, lung cancer incidence was less than predicted among former smokers in the WTC exposed participants. This may reflect limitations in prediction performance for former smokers with the PLCO model or imprecision in self‐reported smoking quit duration.^21^ Additionally, occupational cohort studies have often described a “healthy worker” effect or bias where cancer incidence rates are less than expected.^10^ Although this effect may still exist in the WTC responder cohort, tobacco smoking rates were sufficient in this large cohort to support the implementation of accepted CT‐based lung cancer screening in this population. The overall harms and benefits of lung cancer screening need more detailed assessment in this group employing larger studies of lung cancer risk,^22^ evaluations of screening complications as well as overall life expectancy for the cohort. Comprehensive assessment using empirical data and simulation models may support expanded screening criteria for some patient groups, such as those with mineral dust or other high‐risk pre‐WTC occupational exposures.

Our study had several strengths and weaknesses. It benefited from prospective data with detailed baseline data collection from a well‐described WTC RRWV cohort. We adjudicated all lung cancer cases by using the electronic health records from a large health system that has provided the majority of the health care for this sample. In addition, WTCHP healthcare coverage for lung cancers enhances the likelihood that cases would be reported to the clinical center regardless of where the cancers were diagnosed. To estimate the contribution of unique exposures for the WTC RRWV population we used a novel comparator group, arguably superior to using population‐based data, to estimate the comparative lung cancer risk for the WTC general responder cohort. Our study was limited, however, by a lack of inclusion of longitudinal data on smoking behaviors and by some missing data on baseline characteristics.

In a large, prospective cohort of WTC general responders, we found rates of lung cancer incidence similar to expectation, based on the individual risk profiles of the cohort participants. We find no evidence of WTC exposure contributing a substantial risk increase for lung cancer, although many WTC responders may be at increased lung cancer risk due to traditional risk factors as well as occupational exposures. Notably, though, the cohort is just entering the period following a 20‐year latency from exposure to WTC‐related dust and smoke. Lung cancer surveillance and periodic evaluations of population‐level lung cancer risk should continue in this group.

## CONFLICT OF INTEREST

Dr. Wisnivesky has received consulting honorarium from Sanofi, Glaxosmithkline, and Banook and research grants from Sanofi and Quorum. The other authors report no conflicts.

## AUTHOR CONTRIBUTIONS

*Study conception and design*: KS and JPW. *Acquisition, analysis, and interpretation of data*: All authors. *Critical revision for important intellectual content*: All authors. *Statistical analysis*: KS and JPW, CYK. *Obtained funding*: KS and JPW. *Administrative, technical, or material support*: KS and JPW. *Study supervision*: KS. All authors approved the final version of the manuscript before submission.

## ETHICS STATEMENT

This study was approved by the Mount Sinai Program for the Protection of Human Subjects (HS18‐00583).

## Supporting information


DataS 1

## Data Availability

The data that support the findings of this study are available from the WTCHP Data Center. Restrictions apply to the availability of these data, which were used under license for this study. Data are available from the authors with the permission of the WTCHP.
