# Supplementary material for: Lung cancer incidence among world trade center rescue and recovery workers
Source: Cancer Med. 2022 Mar 28;11(16):3136–44. doi: 10.1002/cam4.4672 (PMC9385594; doi:10.1002/cam4.4672)
Supplement: Supplementary file 1 — DataS 1 [file CAM4-11-3136-s001.docx]

| **Table S1.** Associations of other predictors from multivariable models | | |
| --- | --- | --- |
| **Characteristic** | **Incidence Rate Ratio** | **95% CI** |
| **Obstructive lung disease*** | 1.97 | 1.04-3.75 |
| **Occupational Exposures** |  |  |
| Asbestos | 0.82 | 0.42-1.60 |
| Cadmium | 2.11 | 0.76-5.90 |
| Diesel fumes | 0.99 | 0.60-1.63 |
| Non-diesel industrial fumes | 0.59 | 0.30-1.16 |
| General dust exposure | 0.86 | 0.52-1.42 |
| Mineral dust | 2.03 | 1.07-3.86 |
| Wood dust | 1.21 | 0.70-2.10 |
| Silica dust | 1.31 | 0.73-2.34 |
| Fiberglass | 1.20 | 0.64-2.30 |
| Industrial | 1.17 | 0.65-2.11 |
| Welding | 1.10 | 0.56-2.16 |
| *defined by FEV1/FVC < 70.  Each row represents an individual adjusted model, with additional covariates of age, race/ethnicity, educational attainment, sex, pack-year smoking, years since quite smoking for former smokers and categorical WTC exposure. | | |

| **Table S2.** Multivariable model used to predict missing body mass index values | | |
| --- | --- | --- |
| **Characteristic** | **Coefficient** | **p-value** |
|  |  |  |
| **Age** | 0.03 | <0.001 |
| **Female sex** | -1.81 | <0.001 |
| **Race/ethnicity** |  |  |
| White | Reference |  |
| Black | 0.72 | <0.001 |
| Hispanic | -0.05 | 0.60 |
| Multiracial | -2.07 | <0.001 |
| Other | 1.06 | <0.001 |
| **Education** |  |  |
| <High school | Reference |  |
| High school graduate | 0.79 | <0.001 |
| Some college | 0.93 | <0.001 |
| College graduate | -0.21 | 0.26 |
| Graduate school | 0.38 | 0.11 |
| **Pack-years smoking** | -0.001 | 0.72 |
| **Chronic obstructive pulmonary disease** | 0.26 | 0.01 |
